# Assessing the Current Landscape of Reptile Pet Ownership in Hong Kong: A Foundation for Improved Animal Welfare and Future Research Directions

**DOI:** 10.3390/ani14121767

**Published:** 2024-06-12

**Authors:** Sze-Wing Chan, Wei-Ta Fang, Ben LePage, Shun-Mei Wang

**Affiliations:** Graduate Institute of Sustainability Management and Environmental Education, National Taiwan Normal University, Taipei 106, Taiwan; wtfang@ntnu.edu.tw (W.-T.F.); benlepage2@ntnu.edu.tw (B.L.); t73004@ntnu.edu.tw (S.-M.W.)

**Keywords:** Hong Kong, reptile pet trends, reptile pet ownership, reptile pets, reptile welfare

## Abstract

**Simple Summary:**

In this study we examined reptile pet ownership trends and animal welfare practices in Hong Kong. Over 200 owners from Hong Kong were surveyed and their responses were then used to understand the current preferences for reptile species and how these animals are cared for. The results revealed a growing popularity for reptiles, particularly lizards, among people that were acquiring their first pet reptile. Turtles remained a popular choice, likely due to cultural associations and perceived ease of care. While many owners expressed awareness of proper reptile care, which includes healthcare, diet, and living space, a concerning gap was identified between reptile care knowledge and its practical application. This was particularly evident in the under-utilization of veterinary check-ups. Interestingly, we found a potential link between the experience of owning a pet reptile and reptile health, suggesting new owners might benefit from acquiring more knowledge. Additionally, we identified a possible association between the variety of sources from which reptiles are acquired and their well-being. These findings highlight the need for more focused educational programs to equip new reptile pet owners with proper health care and nutrition practices and the importance of regular veterinary visits. By fostering collaborations and/or partnerships between reptile pet owners, veterinarians, and animal welfare organizations, the results of this study and their application aim to improve reptile welfare within the evolving pet ownership landscape in Hong Kong.

**Abstract:**

We investigated the evolving landscape of reptile pet ownership in Hong Kong. Employing a quantitative approach, a self-administered survey was distributed and over 200 reptile pet owners residing in Hong Kong responded. The survey instrument captured demographic data on pet ownership history, species preferences, husbandry practices, and veterinary care utilization. The findings revealed a strong interest in pet reptiles, with lizards being particularly popular (67%) among new owners. Turtles remain common (35%), likely due to cultural factors and perceived ease of care. However, a gap was noted between the awareness and the implementation of proper husbandry practices, with 51% of respondents expressing concerns about enrichment and 21% uncertain about appropriate enclosure size. Veterinary care utilization also showed a disconnect, with 50% of the respondents reporting no regular check-ups were performed, despite acknowledging its importance. Reptile behavior served as a well-being indicator. Over 90% of the respondents observed normal behaviors like locomotion and breathing. Interestingly, a positive correlation emerged between reptile behavior scores and duration of ownership (r = 0.200, *p* < 0.01), suggesting improved well-being for reptiles that were in long-term care. These findings emphasize the need for educational initiatives promoting responsible pet ownership practices and fostering collaboration between reptile owners, veterinarians, and animal welfare organizations. By addressing these knowledge gaps and promoting a collaborative approach, our results aim to contribute to enhanced reptile welfare in the context of Hong Kong’s evolving pet ownership trends.

## 1. Introduction

The global trade of reptiles in the pet industry has emerged as a significant threat to their conservation and welfare [1]. Over one-third of all known reptile species are actively being bought and sold, raising serious ethical and ecological concerns [2]. The supply chain complexity encompasses sourcing, transportation, and their health care in a variety of spatial and temporal settings. These issues subject reptiles or any animal to a multitude of health and well-being challenges throughout its journey. Wild-caught individuals constitute roughly half of the trade, and endure immense stress and suffering due to capture, habitat disruption, confinement, and exposure to unfamiliar environments and predators [2,3,4]. Even captive-bred reptiles can face unintended genetic disorders from selective breeding practices [5]. During transport, reptiles experience noise, vibration, temperature fluctuations, and inadequate lighting, leading to compromised health and even death [6,7]. Upon arrival at pet stores or expositions, inadequate housing often restricts their natural behaviors, further compromising their well-being, as highlighted by research on ball python enclosures [8]. These cumulative threats necessitate urgent attention and action to ensure the responsible and humane treatment of reptiles in the global pet trade.

Further complicating these issues, reptile husbandry practices demand expertise that is distinct from the care of traditional companion animals like dogs and cats. Research consistently reveals that many reptile owners inadvertently fail to meet their pet’s essential needs, with deficiencies often present in areas like temperature control, lighting, diet, and appropriate enclosure size [9,10,11]. Beyond the unmet needs, traditional herpetological husbandry practices themselves can raise ethical concerns. Often rooted in tradition rather than evidence-based principles, these practices may lack scientific justification and even contradict contemporary herpetological research data [12]. Such shortcomings can expose captive reptiles to chronic stress, leading to injuries, illnesses, and compromised welfare [13,14,15].

In the context of Hong Kong, understanding the reptile pet trade and its associated welfare implications is hampered by a lack of current and comprehensive data. The existing information on exotic pets is primarily limited to turtles (encompassing turtles and tortoises) and dates back to 2005 [16], significantly lagging behind the current trends. While data on overall household reptile pet ownership remain scarce, anecdotal evidence suggests a surge in their popularity. Statistics reveal a doubling of non-traditional pet imports (excluding common companion animals) between 2012 and 2017, with the top five reptile species consistently representing 55–70% of this increasing demand [17]. This growing interest, however, is overshadowed by concerns around the release or escape of non-native reptile species into the local environment [18,19,20], further highlighting the need for responsible pet ownership and management strategies.

Given the scarcity of current and comprehensive data, a thorough investigation into the contemporary state of the household reptile pet market in Hong Kong is urgently needed. This study aims to address this gap by investigating key aspects, including the demographics of both reptile owners and the traded species, the husbandry practices currently employed by owners, and their perspectives on animal welfare for their reptile companions. By examining these factors alongside reptile behaviors, we can gain valuable insight into potential welfare concerns associated with current trends in the Hong Kong reptile pet market. Armed with this knowledge, stakeholders can develop targeted interventions and educational initiatives to ensure the well-being of captive reptiles and promote responsible pet ownership practices.

## 2. Materials and Methods

### 2.1. Survey Development

Drawing from a literature review focusing on reptile pet ownership and welfare, an anonymous online survey was designed using Google Forms to collect data from the current owners of snakes, lizards, and turtles in Hong Kong. This focus on these specific reptile species aligned with the regulations outlined in Reg. 5(3) of the Public Health (Animals and Birds) (Trading and Breeding) Regulations (Cap. 139B) in Hong Kong. These regulations prohibit the trade of certain species, including crocodiles, alligators, and tuatara. Consequently, the survey concentrated on the most commonly encountered reptiles in the Hong Kong pet market: snakes, lizards, and turtles.

The survey consisted of three sections, targeting current reptile owners and aiming to collect information on their practices. The first part, owner background, collected data on respondent demographics and pet-keeping history to understand the owner’s experience level and characteristics.

The second part, husbandry practices, focused on collecting data on the owner’s reptile care routines. This section was further divided into two subsections. The self-assessment subsection was comprised of seven questions. Two general questions gauged the owner’s perceptions of whether they were fulfilling their reptile’s physical needs and providing an “as close as possible” natural environment. The remaining five questions addressed specific husbandry aspects such as: enclosure size, provision of appropriate basking areas, meeting temperature requirements, offering a diversified diet, and ensuring access to proper veterinary care. The owners rated their practices on a Likert scale that ranged from 1—disagree to 4—agree. The actual practices subsection offered owners multiple options to indicate their specific husbandry practices in providing light sources, temperature control mechanisms, hiding spaces, food options/diversity and frequency of veterinary check-ups. Additionally, it explored how owners typically dealt with a sick reptile.

The six questions in the final section, pet behaviors, were aimed to identify any abnormal behaviors displayed by the reptiles. These questions were designed to explore issues related to locomotor activity, interaction with enclosure walls, basking patterns, respiration, responses towards humans, and actions displayed in response to human presence or handling.

The survey instrument was adapted and modified from the published literature to ensure content validity and reliability. The owner background and self-assessment items were adapted from the work of Huang [21], while the framework for reptile welfare was adopted from Warwick et al. and Azevedo et al.’s work [10,22], with modifications for the general application to turtles, lizards, and snakes. To enhance the quality of the survey, the questions were reviewed by four experts and professors specialized in environmental education, animal welfare, and amphibian biology and herpetology. Their valuable insight and feedback were incorporated into the questions prior to finalizing the survey.

The survey was disseminated across various social networking platforms (including WhatsApp, Line, Signal, Facebook, and Instagram) during a period spanning from 10 March 2022, to 11 April 2022. Upon initiating the survey, participants encountered an informed consent statement outlining the study’s objective of gaining a deeper understanding of pet reptile husbandry practices. Individuals who provided their consent proceeded to the survey, while those who declined were unable to continue. The full questionnaire is provided in Table S1 in the Supplementary Materials.

### 2.2. Data Preparation and Refinement

The refinement of the survey responses was conducted in Microsoft Excel to address two key inconsistencies. Inconsistencies in responses to the question “Do you currently keep pet(s) other than reptiles?” in Part 1 were addressed. A small number of respondents answered “yes”, but later indicated owning “turtles” or “none” for specific species. These responses were recorded for consistency. This change was made to accurately reflect owning no pets aside from reptiles. Second, four respondents omitted identifying the reptile species in the Part 2 question “The species of your reptile” but confirmed owning only one species from the Part 1 question “Reptile(s) currently owned is/are”. Their species information was imputed based on their responses to the question in Part 1. A total of four instances of missing or ambiguous information for the Part 1 question “At what age did you keep your first reptile?” were excluded from analyses.

Based on the criteria established by Azevedo et al. [10], responses concerning access to light, enclosure temperature, hiding space, and regular check-ups were coded dichotomously. A detailed justification for this scale is provided in Table S2 in the Supplementary Materials. To ensure data accuracy and relevance, responses lacking species identification or containing ‘Don’t know’ responses were excluded for ‘access to light’ and all four practices, respectively, to eliminate potential bias and maintain the integrity of subsequent analyses.

Building on the Warwick et al. and Azevedo et al. approaches [10,22], six reptile behaviors—(i) locomotor activity, (ii) interaction with enclosure walls, (iii) basking, (iv) breathing, (v) actions towards humans and (vi) actions in response to human presence or manipulation—were classified as either “normal” or “abnormal” based on their potential impact on the animal’s welfare. A detailed justification for this categorization is provided in Table S3 in the Supplementary Materials. Normal behaviors that were considered indicative of positive welfare were assigned a score of 1 and summed cumulatively to create a reptile behavior score.

### 2.3. Statistical Analysis

Descriptive statistical approaches, such as the Kruskal–Wallis and Spearman’s correlation tests, were used to shed light on the dynamics of reptilian pet ownership in Hong Kong. This methodology facilitated the development of a foundational understanding and the relationships between reptile pet owner demographics and patterns, husbandry practices, and pet behaviors.

## 3. Results

### 3.1. Demographic Profile

The data recovery rate was 94.9% and yielded 206 valid questionnaires that were completed by Hong Kong residents. Gender distribution was skewed, with nearly double the number being female (n = 136) compared to male (n = 70). Age distribution spanned across multiple generations, ranging from 15 to 64 years, with the largest being the 25–44 age group, representing 65% of respondents (Table 1).

The participant poll was well-educated, with 49% holding degrees and 21% completing non-degree programs, and most were working (Table 1).

Household size mirrored demographic trends and urban living preferences in Hong Kong. The majority (74%) resided in small- to medium-sized households, measuring 100–500 square feet with 2–4 individuals, which is common in Hong Kong. Household income was positively skewed, with over half (55%, n = 114) exceeding the median income of HKD 30,000 in Hong Kong [23]. Notably, 21% (n = 24) earned above HKD 50,000, highlighting a financially stable segment of the population in this study.

### 3.2. Reptile Pet Ownership

#### 3.2.1. Shared Homes and Diverse Collections

**Figure 1:**
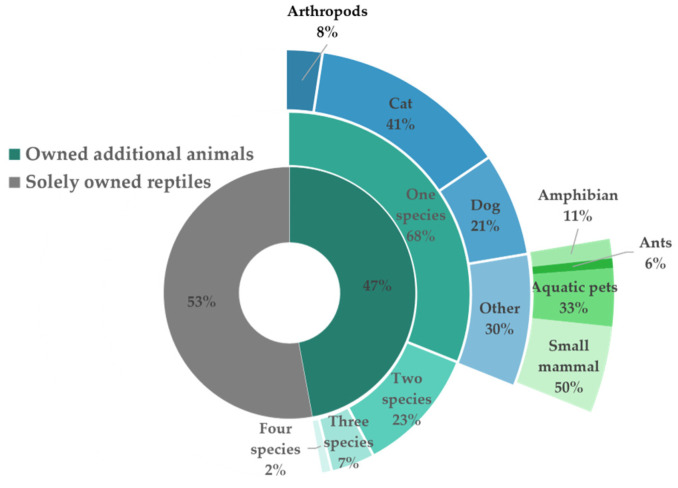
Companion animal diversity among reptile owners: Of the 206 respondents, 97 (47%) kept additional animals beyond their reptilian companions, while 109 (53%) solely owned reptiles. Among the multi-species owners (n = 97), 66 (68%) kept one additional type of animal, 22 (23%) housed two additional types, and a smaller group opted for three (7, 7%) or even four (2, 2%) different species. Among those with one additional pet, cats reign supreme (27, 41%), followed by dogs (14, 21%). A diverse array of other creatures also finds a place in these homes, with a smaller presence of arthropods (5, 8%), amphibians (2, 11%), ants (1, 6%), aquatic pets (6, 33%), and small mammals (9, 50%). These data showcase the varied preferences and personal touches individuals incorporate into their reptile pet ownership experience, highlighting both popular choices and a captivating range of other animal companions.

Table 2 paints a detailed picture of the reptilian pet ownership patterns in Hong Kong. While over half (53%) of the respondents exclusively housed reptilian companions, nearly half (47%) shared their lives with other animals. Cats and dogs were the most popular co-habitants, reflecting widespread preferences for these furry companions (Figure 1). Interestingly, a small but intriguing group maintained diverse multi-species ensembles, showcasing the varied preferences and lifestyles among reptile enthusiasts.

**Figure 2:**
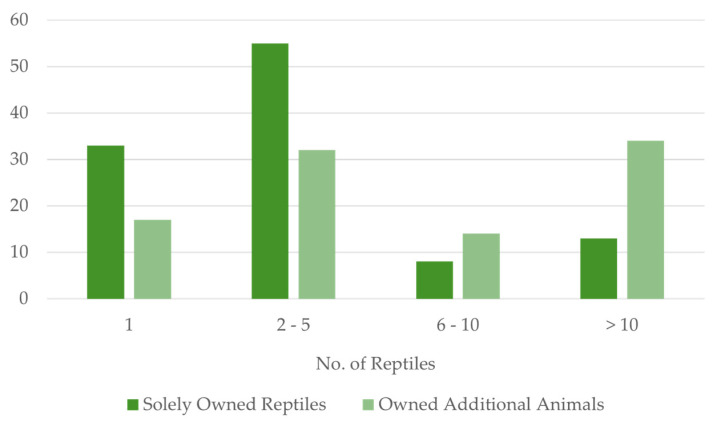
This figure illustrates the distribution of reptile ownership among survey respondents. Of the 206 respondents, 109 (53%) reported owning reptiles only. Within this group, 33 (16%) owned 1 reptile, 55 (27%) owned 2–5 reptiles, 8 (4%) owned 6–10 reptiles, and 13 (6%) owned more than 10 reptiles. Additionally, 97 respondents (47%) reported owning other types of animals alongside reptiles. Among these respondents, 17 (8%) owned 1 reptile, 32 (16%) owned 2–5 reptiles, 14 (7%) owned 6–10 reptiles, and 34 (17%) owned more than 10 reptiles.

Collection size revealed intriguing trends. The majority (67%) maintained small collections of 1–5 reptiles, with 25% owning just one. However, a dedicated minority boasted larger collections exceeding 10 animals, highlighting the diverse spectrum of ownership styles. The results revealed a potential association between the number of reptiles owned and the presence of other animal species in a respondent’s collection (Figure 2). Among those owning 1–5 reptiles (n = 137), over half (88/137) exclusively kept reptiles. However, this pattern shifted for individuals with larger reptile collections (>5 reptiles, n = 69). Here, nearly 70% (48/69) not only owned more than 5 reptiles but also possessed additional animal types. Furthermore, this trend strengthened for those with extensive reptile holdings exceeding 10 reptiles. While 13 individuals in this group solely owned reptiles, a considerably larger number (34) maintained collections encompassing other animal species alongside their reptiles.

#### 3.2.2. Species Diversity

**Figure 3:**
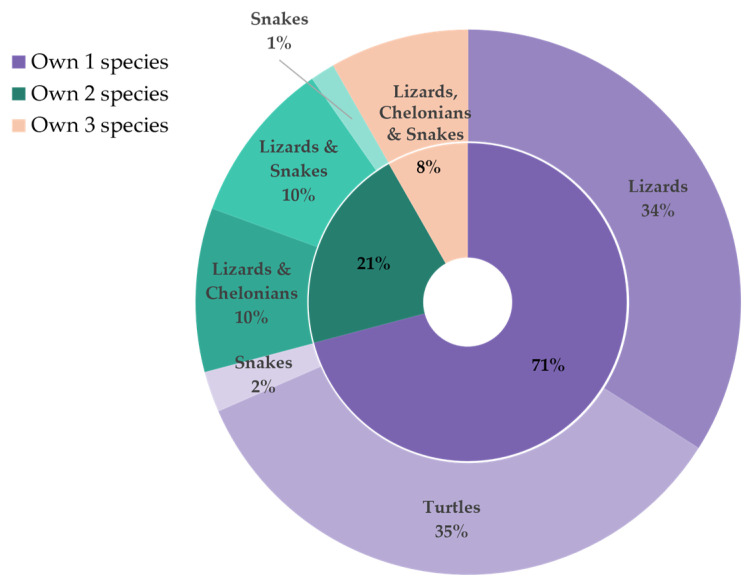
Distribution of respondent-owned reptile species in Hong Kong: The inner ring shows the percentage of respondents keeping a number of species (1, 2, or 3), while the outer ring details the specific species composition of those collections.

Figure 3 unveiled a multifaceted landscape of reptile collections. Over two-thirds (71%) of the respondents had opted for single-species ownership, with lizards (34%) and turtles (35%) representing the most popular choices. Intriguingly, among those that had ventured into multi-species ownership, captivating combinations emerged, with lizard–turtle and lizard–snake pairings each accounting for 10% of the combinations. This finding suggests a possible preference for inter-species compatibility. As expected, a statistically significant positive correlation (r = 0.596, *p* < 0.001) existed between the number of reptiles owned and the number of species represented, indicating that larger collections harbored greater species diversity (Table 3).

#### 3.2.3. Pet Ownership Duration

**Figure 4:**
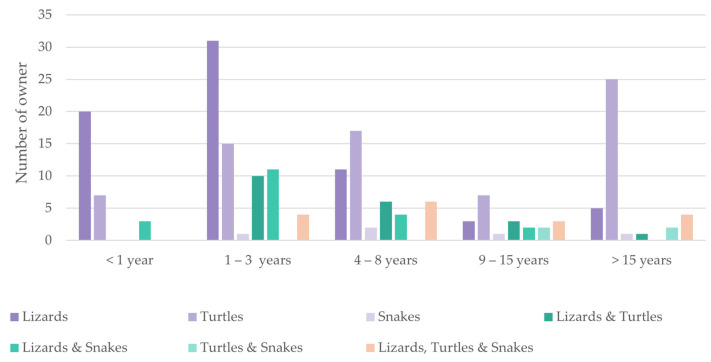
Distribution of reptile species ownership by duration (number of years): The number of respondents owning a species combination over time are presented in Figure 3. Lizards dominated the shorter ownership periods, while turtles became increasingly popular over time. Multi-species ownership was present across all animal categories, although snakes were less frequently owned.

Initial reptile pet ownership predominantly occurred during childhood or young adulthood, with nearly half (47%, n = 96) of the respondents acquiring their first reptile before the age of 20 (Table 2). This suggests early exposure and a lasting fascination with these creatures. However, the duration (years) of these reptile–human bonds varied considerably (Figure 4). While some individuals experienced short-term interactions, others cultivated long-lasting connections spanning over 15 years. Curiously, these varying durations revealed distinct species preferences: lizards dominated short-term ownership, while turtles emerged as the preferred choice for long-term ownership. Furthermore, an inverse relationship emerged between acquisition age and pet ownership duration (r = −0.499, *p* < 0.001) (Table 3). Individuals who acquired their first reptile earlier in life exhibited longer ownership durations, potentially reflecting that deeper inter-species connections were established early on.

#### 3.2.4. Reptile Acquisition Methods

**Figure 5:**
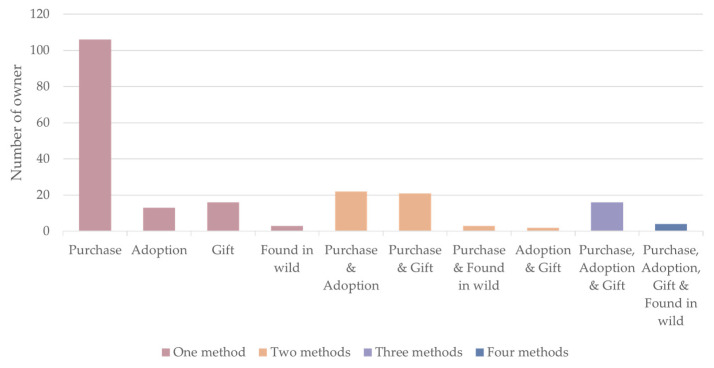
Acquisition method: This figure illustrates the diverse acquisition methods employed by the 206 respondents. Among the respondents, single acquisition methods (138) were most common, led by purchase (106). Adoption (13), gifting (16), and finding in the wild (3) followed. While 33% (68) utilized multiple methods, purchase and adoption (22, 11%), purchase and gift (21, 10%), and purchase, adoption and gift (16, 8%) were more popular than other combinations.

Figure 5 illuminates the varied means through which individuals acquired reptiles. Acquisition primarily occurs through purchase, favored by the majority (67%) of respondents. Other avenues included adoption (6%), gifts (8%), and encountering and acquiring animals in the wild (1%). A statistically significant positive correlation emerged between the acquisition methods employed and the number of reptiles owned (r = 0.401, *p* < 0.001) and the number of species represented (r = 0.385, *p* < 0.001) (Table 3). This indicates that individuals with larger and more diverse collections tend to utilize a wider range of acquisition methods.

#### 3.2.5. Motivations for Reptile Pet Ownership

Delving deeper, our open-ended responses unveiled seven key motivations: adoption, aesthetic appeal, ease of care expectations, educational value, emotional connection, family/friend influence, and “other” (Table 4). Beauty and emotional connection emerged as dominant factors (77 and 74 mentions, respectively), suggesting a deep appreciation for these animals’ unique visual appeal and capacity for companionship. Ease of care (38 mentions) and external influences from family and friends (22 mentions) also played important roles in the decision-making process, while adoption and educational value held less influence, but were motivations for some. This nuanced exploration underscores the multifaceted nature of reptile pet ownership, driven by a combination of personal preferences, societal influences, and individual values.

#### 3.2.6. Satisfaction with Reptile Pet Ownership

Overwhelmingly, 97% of respondents reported positive experiences with reptile pet ownership with minimal disagreement (Table 2). This finding suggests a broad appeal and a perception of the beneficial interactions with these creatures, highlighting the potential for enriching and fulfilling human–animal bonds.

Individuals with larger and more diverse collections, and those who acquired their reptiles through various methods, reported marginally higher levels of satisfaction, with positive correlations between satisfaction and the number of reptiles owned (r = 0.143, *p* < 0.05), number of species (r = 0.184, *p* < 0.01), and number of acquisition methods (r = 0.177, *p* < 0.05) (Table 3). However, contrary to expectations, a negative correlation emerged between satisfaction and duration of ownership (r = −0.181, *p* < 0.01), indicating that longer ownership was associated with slightly lower satisfaction levels (Table 3).

#### 3.2.7. Gender Differences in Reptile Pet Ownership

The Kruskal–Wallis test analysis revealed statistically significant gender differences in several aspects of reptile pet ownership (*p* < 0.05) (Table 5). Males reported owning a greater number of reptiles and a wider variety of species compared to females (*p* = 0.024 and *p* = 0.033, respectively). Additionally, males acquired their first reptile at a younger age and enjoyed longer durations of reptilian companionship compared to females (*p* = 0.008 and *p* ≤ 0.001, respectively). These findings suggest potential gender disparities in reptile pet ownership patterns and experiences.

### 3.3. Husbandry Practices and Reptile Welfare

Although the data in Table 6 indicated awareness of the importance of general husbandry practices, concerns surfaced regarding environmental enrichment and enclosure size. Only 51% of respondents felt their reptiles thrived in natural-like habitats, suggesting potential shortcomings in enrichment strategies. This underscores the importance of providing stimulating environments that cater to the reptile’s innate behaviors and needs, promoting physical and mental well-being. Apart from that, 21% of the respondents expressed concerns about inadequate enclosure size, prompting further investigation into optimal enclosure dimensions for various reptile species.

**Figure 6:**
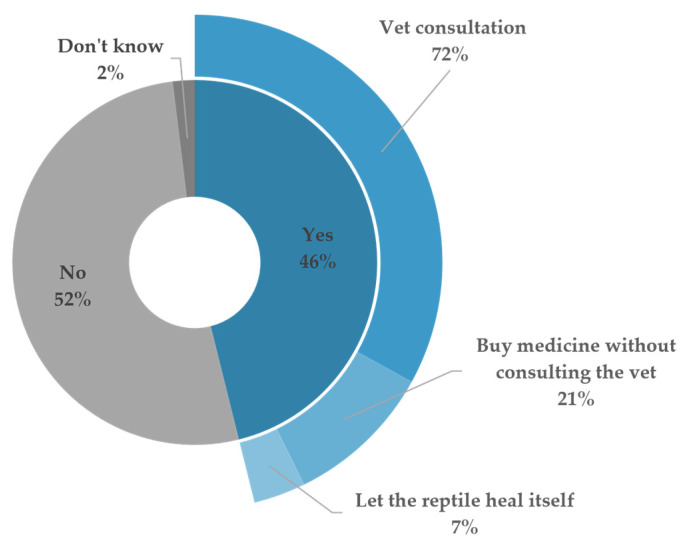
The distribution of reported past illnesses in reptiles and owner-chosen intervention methods. The inner ring illustrates the percentage of respondents reporting past illnesses, while the outer ring details the proportion of owners choosing different intervention methods.

There was agreement on the importance of proper medical care (Table 6), but a disconnect between perceived value and actual practices (Table 7). Fifty percent of the respondents reported no regular veterinary check-ups, indicating a gap between awareness and action. On the other hand, among the reptiles with reported illnesses, 72% received veterinary consultation, while 21% self-medicated, and 7% employed non-interventionist approaches (Figure 6). These responses underscore the potential need for further guidance and/or education on animal health management.

An analysis of dietary habits revealed a preference for multi-component diets that were aligned with recommendations for balanced nutrition. Live food emerged as the most popular choice (141 instances), followed by commercial feed (125), vegetables (115), and fruits (95). Only a small fraction (14) solely relied on commercial feed, while a substantial number of owners (47) included live food in their reptile’s diets. This suggests an awareness of the importance of dietary diversity in meeting reptiles’ specific nutritional requirements.

### 3.4. Reptile Behavior and Well-Being

Reptile behavior was an important indicator of an animal’s well-being, including a significant decrease in activity level, unusual interactions with their enclosure walls, or a loss of appetite. All can be signs of potential health concerns [22]. The results of this study offered encouraging results. Over 90% of the respondents observed normal locomotion (92%) and breathing (91%) patterns (Table 8). Similarly, low frequencies of aggression towards humans (86%) and stress-related responses (85%) were reported. While the frequency of basking behavior (78%) and interaction with enclosure walls (70%) were slightly lower, the overall findings suggest a generally positive perception of reptile well-being, with most animals exhibiting expected behaviors.

A number of statistically significant correlations between reptile behavior scores and husbandry practices emerged (Table 9). There was a positive correlation (r = 0.139 (*p* < 0.05) between reptile behavior and owner living space area. This suggests reptiles housed in larger enclosures tend to exhibit more positive behaviors. Additionally, a strong positive correlation (r = 0.200, *p* < 0.01) was observed between reptile behavior score and duration of ownership, indicating that reptiles owned for longer durations tended to have higher scores. Furthermore, a weaker but significant positive correlation emerged linking the reptile behavior score to the number of acquisition methods employed (r = 0.162, *p* < 0.05), suggesting that individuals utilizing diverse acquisition methods also reported reptiles with higher scores.

## 4. Discussion

### 4.1. Limitations

Despite achieving a high data recovery rate, limitations related to online survey methodology are acknowledged. First, there is a potential skewing towards younger female respondents. Although Hong Kong enjoys a high internet penetration rate, official data reveal a significantly lower usage among individuals aged 65 and above [24]. This potential exclusion of older people is further compounded by research indicating a higher concentration of social media users in the under 45 age and female groups [25]. These factors align with the observed under-representation of older respondents and potential skewing of results towards younger females in the study sample. The findings of this study provide valuable insight into the landscape of reptile ownership in Hong Kong. However, future studies employing larger and more representative samples across different demographic variables, particularly with increased representation from older age groups, would be necessary to confirm these findings and enhance their generalizability to the entire reptile-owning population within the city.

Second, the ability to capture the full spectrum of reptile welfare concerns was limited by the questionnaire’s focus on questions that were applicable to turtles, lizards, and snakes. The well-documented inter-species variation in reptile welfare necessitates species-specific assessment approaches [10]. The diverse reptile species present in Hong Kong necessitate such tailored assessments, which were hindered by the lack of data on ownership proportions and the prevalence of multi-species households.

### 4.2. Reptile Preferences in Hong Kong

This study delved into the fascinating world of reptile pet ownership in Hong Kong, revealing trends that align and diverge from global patterns. Similar to a global phenomenon, the study highlights a growing preference for lizards among new reptile owners in the city [26]. Lizards are often perceived as visually striking and interactive creatures and seem to capture the hearts of first-time reptile enthusiasts in Hong Kong. This trend mirrors observations in other parts of the world, where lizards are increasingly gaining popularity as captivating pets [26].

However, Hong Kong also displays a unique cultural influence on reptile pet ownership. Turtles continue to enjoy enduring popularity, which is likely due to two key factors. First, traditional Chinese culture often associates turtles with longevity, imbuing them with a symbolic significance that transcends their role as pets [27]. Second, turtles are often perceived as requiring minimal care, making them attractive options for busy individuals seeking low-maintenance companions [27]. This aligns with the observation that owners with extensive experience, often exceeding 15 years of ownership, frequently have turtles in their collection. This suggests that the perceived ease of caring for turtles might be particularly appealing to long-term reptile owners who may have evolving needs and preferences.

Intriguingly, the study revealed a contrasting trend when compared to Western countries. While snakes hold a prominent position in the reptile pet trade of the West [28,29], they appear to be less popular among the general public in Hong Kong. However, this observation seems to contradict another interesting fact where Hong Kong ranks as the second-largest importer of ball pythons from West Africa [30]. This apparent discrepancy warrants further investigation. Two potential explanations emerge. First, Hong Kong might primarily function as a re-export hub for ball pythons, acting as a middle ground for the global trade for these popular snakes. Ball pythons imported to Hong Kong could then be distributed to other countries with a higher demand for these reptiles.

Second, it is possible that a sub-population of dedicated snake enthusiasts exists in Hong Kong. These individuals might maintain large personal collections, skewing the overall ownership numbers towards a smaller segment of specialized snake owners. This scenario could explain the high volume of imported ball pythons despite the seemingly lower overall popularity of snakes among the public in Hong Kong.

Further research is needed to definitively uncover the reasons behind this intriguing contrast. Understanding the dynamics of the reptile trade in Hong Kong, along with the motivations of dedicated snake enthusiasts within the city, will be crucial in creating a more comprehensive picture of reptile pet ownership trends in this unique cultural landscape.

### 4.3. Gap between Awareness and Action in Veterinary Care

A concerning disconnect exists between reptile owner awareness and veterinary care utilization. While many owners believed their scaly companions receive proper medical attention, only 50% brought their reptiles for regular check-ups. This significant gap highlights a critical need to understand the underlying reasons behind this behavior. Veterinary care is a cornerstone of animal welfare [31]. Owners have a fundamental responsibility to ensure their pets receive preventive healthcare, including disease prevention, timely diagnoses, and effective treatment when illness strikes. This is especially crucial for reptiles, whose evolutionary adaptations often lead them to mask signs of illness [32]. A thorough examination by a veterinarian may be the only opportunity to identify potential problems early on, allowing for interventions that improve treatment outcomes and overall well-being.

Understanding this gap in veterinary care utilization is crucial. Reptile owners’ underutilization of veterinary care stems from a confluence of factors. These include misconceptions about reptile longevity and healthcare needs, along with a perceived lack of qualified reptile veterinarians [33]. Veterinarians that are also properly trained as reptile veterinarians may be limited to those that are trained to treat cats and dogs. By identifying the primary drivers of this behavior, we can develop targeted interventions to improve reptile welfare. Educational initiatives aimed at highlighting the importance of regular veterinary check-ups and dispelling misconceptions about reptile healthcare needs can be instrumental in closing this awareness–action gap. Additionally, promoting the availability of qualified reptile veterinarians and fostering a collaborative relationship between owners and veterinary professionals can ensure reptiles receive the care they deserve.

By addressing the awareness–action gap, we can pave the way for a future where reptile pet ownership prioritizes the health and well-being of their pets. Through a combination of education, increased access to qualified veterinarians, and collaborative relationships, we can ensure that reptiles receive the veterinary care they deserve, allowing them to thrive as cherished members of our households.

### 4.4. Links between Experience, Acquisition Methods, and Behavior

We examined the fascinating connections between humans and reptile pet ownership, acquisition methods, and the overall behavioral health of these captive animals. The findings show a positive correlation between pet ownership duration and reptile behavior scores, suggesting that longer periods of pet ownership likely translate to increased knowledge and expertise gained through experience [34]. This positive correlation underscores the critical importance of promoting responsible husbandry practices and readily available knowledge resources for new reptile pet owners. Imagine a world where new reptile enthusiasts have easy access to comprehensive guides on proper care, temperature control, diet composition, and enclosure design. Such resources can empower them to provide their reptilian companions with optimal living conditions, contributing significantly to their well-being.

We further took an even more intriguing turn by identifying a potential link between reptile behavioral health and the variety of acquisition methods employed by owners. Our findings hint at the potential benefits of a wider support network for people that acquire reptiles through diverse sources. This network could encompass a rich tapestry of individuals and organizations, including experienced reptile owners who can offer practical advice, reputable breeders who prioritize ethical sourcing and proper care, and dedicated animal welfare organizations that provide guidance on responsible reptile pet ownership. However, we acknowledge the need for further research to definitively determine the nature and extent of this influence. Investigating how these support systems function and the specific ways they contribute to improved reptile welfare would be a valuable next step. Unveiling the intricate workings of these support systems would provide valuable insights for promoting positive interactions between humans and reptiles in captivity.

By delving deeper into the connections between pet ownership experience, acquisition methods, and reptile behavior, more effective strategies can be developed to enhance the well-being of reptiles in captivity. Educational initiatives and readily accessible resources can equip new owners with the knowledge necessary to provide proper care, while fostering connections within the reptile owner community and facilitating communication with animal welfare organizations can create a supportive network for even the most experienced reptile enthusiasts. Furthermore, a focus on responsible reptile care can benefit stakeholders throughout the industry. By educating pet owners about the specific needs of different species, such as the requirement for both freshwater and saltwater systems for certain turtles, the aquarium trade can also flourish as the demand for appropriate habitats increases. This comprehensive approach can benefit all stakeholders involved in the reptile pet world, ensuring the well-being of these fascinating creatures.

### 4.5. Enhancing Reptile Welfare through Education: Future Directions in Hong Kong

The findings from this study, particularly the concerns regarding husbandry practices and the disconnect between the perceived value and actual utilization of veterinary care, highlight the potential knowledge gaps among Hong Kong reptile owners. To address these issues and improve reptile welfare in Hong Kong, future research should delve deeper into the reasons behind these phenomena within the specific context of the people and culture of Hong Kong residents and reptile ownership.

Once a clearer understanding of the underlying causes is established, the development and implementation of targeted educational programs specifically tailored to the needs of Hong Kong reptile owners can be explored. For example, if knowledge gaps are identified in areas like reptile behavior, educational interventions could be delivered by animal welfare organizations. These interventions could take the form of courses, talks, or informative materials distributed through local pet shops and social media platforms that are popular among Hong Kong reptile enthusiasts.

In addition to reptile-specific knowledge, education on positive animal welfare principles could be beneficial. This approach focuses on ensuring animals have the opportunity to experience well-being, which could motivate Hong Kong reptile owners to strive for even higher welfare standards for their pets [35].

## 5. Conclusions

We investigated reptile pet ownership in Hong Kong based on the responses provided by over 200 participants, revealing a diversity of demographic profiles and animal ownership. Female ownership dominated, with 66% being female compared to 34% male. Age distribution spanned multiple generations, with the largest concentration falling within the 25–44 age group (65%). This suggests that reptile pet ownership in Hong Kong is prevalent among younger adults and may be associated with changing pet preferences. The growing trend of keeping reptiles as pets in Hong Kong, with its opportunities and challenges, necessitates a closer look at animal welfare. The responses revealed a diverse range of species being kept as companions, with lizards becoming increasingly popular among new pet owners. Turtles retained their appeal due to perceived longevity and ease of care. Concerns surfaced regarding husbandry practices and veterinary care utilization.

Despite acknowledging the importance of proper housing and enrichment, many owners reported shortcomings in these areas. A significant disconnect also existed between the perceived value of veterinary care and its actual utilization. Educational initiatives could likely help to bridge this gap by emphasizing responsible husbandry practices and the critical role of regular veterinary check-ups in maintaining reptile well-being. Promising avenues for improving reptile welfare in Hong Kong were identified. Pet ownership experience appears to positively influence reptile behavior, suggesting that knowledge gained through experience plays a significant role. Interestingly, the variety of acquisition methods employed by owners might also be linked to reptile well-being. Owners who acquired their reptiles through diverse sources, such as breeders, shelters, or other owners, might benefit from a wider support network offering valuable information and guidance. Further research is needed to explore this potential influence in greater detail. A prime example of the potential benefits of responsible pet ownership lies in the critically endangered Chinese Three-striped Box Turtle. While wild capture is now illegal in Hong Kong, existing pet turtles could potentially contribute to conservation efforts. Collaboration between government and conservation organizations could facilitate the surrender of these pet turtles for captive breeding programs, offering a valuable resource for bolstering wild populations. By fostering a supportive network for reptile owners and promoting responsible pet ownership practices through education, Hong Kong can ensure that these captivating creatures not only thrive in captivity but become cherished members of the city’s households for generations to come. This collaborative approach involving researchers, veterinarians, reptile enthusiasts, and policymakers can pave the way for a future where both ethical considerations and the well-being of reptiles are prioritized.

## Figures and Tables

**Table 1:** Reptile owner demographics by gender.

		Total
		n (%)
Gender	Female	136 (66%)
	Male	70 (34%)
Age	<15	15 (7.3)
	15–24	48 (23.3)
	25–44	133 (64.6)
	45–64	10 (4.9)
Educational Level	Junior secondary school	12 (5.8)
	Senior secondary school	50 (24.3)
	Tertiary (non-degree)	44 (21.4)
	Tertiary (Degree)	100 (48.5)
Working Status	Studying	38 (18.4)
	Working	158 (76.7)
	Unemployed	9 (4.4)
	Retired	1 (0.5)
Household Size	1	17 (8.3)
	2–4	165 (80.1)
	5–7	22 (10.7)
	≥8	2 (1.0)
Living space area (sq. ft.)	<100	5 (2.4)
	100–300	36 (17.5)
	301–500	81 (39.3)
	501–800	62 (30.1)
	>800	22 (10.7)
Monthly Household Income	<HKD10,000	11 (5.3)
	HKD10,000–19,999	36 (17.5)
	HKD20,000–29,999	45 (21.8)
	HKD30,000–39,999	40 (19.4)
	HKD40,000–49,999	30 (14.6)
	≥HKD50,000	44 (21.4)

**Table 2:** Reptile pet ownership characteristics by gender.

		Total	Gender	
			Female	Male
		n (%)	n (%)	n (%)
		206 (100)	136 (66.0)	70 (34.0)
Number of pet reptile	1	50 (24.3)	34 (16.5)	16 (7.8)
	2–5	87 (42.2)	66 (32.0)	21 (10.2)
	6–10	22 (10.7)	13 (6.3)	9 (4.4)
	>10	47 (22.8)	23 (11.2)	24 (11.7)
Number of reptile species owned	One species	146 (70.9)	102 (49.5)	44 (21.4)
	Two species	43 (20.9)	28 (13.6)	15 (7.3)
	Three species	17 (8.3)	6 (2.9)	11 (5.3)
Acquired the first reptile at age	1–9	35 (17.0)	20 (9.7)	15 (7.3)
	10–19	61 (29.6)	33 (16.0)	28 (13.6)
	20–29	63 (30.6)	46 (22.3)	17 (8.3)
	30–39	33 (16.0)	26 (12.6)	7 (3.4)
	40–45	10 (4.9)	8 (3.9)	2 (1.0)
	Missing	4 (1.9)	3 (1.5)	1 (0.5)
Reptile pet ownership duration	<1 year	30 (14.6)	29 (14.1)	1 (0.5)
	1–3 years	72 (35.0)	46 (22.3)	26 (12.6)
	4–8 years	46 (22.3)	31 (15.0)	15 (7.3)
	9–15 years	21 (10.2)	9 (4.4)	12 (5.8)
	>15 years	37 (18.0)	21 (10.2)	16 (7.8)
Number of acquisition methods	One method	138 (67.0)	95 (46.1)	43 (20.9)
	Two methods	48 (23.3)	30 (14.6)	18 (8.7)
	Three methods	16 (7.8)	9 (4.4)	7 (3.4)
	Four methods	4 (1.9)	2 (1.0)	2 (1.0)
Satisfied on keeping reptile	1 Disagree	1 (0.5)	0 (0.0)	1 (0.5)
	2 Somewhat Disagree	5 (2.4)	4 (1.9)	1 (0.5)
	3 Somewhat Agree	30 (14.6)	20 (9.7)	10 (4.9)
	4 Agree	170 (82.5)	112 (54.4)	58 (28.2)

**Table 3:** Spearman’s rho correlation coefficients for reptile pet ownership ordinal variables. Statistically significant correlations (*p* < 0.05) are indicated by bold font.

Dependent Variable	Independent Variables	Correlation Coefficient (*r)*	*p* Value	95% Confidence Intervals	
				Lower	Upper
Number of pet reptiles	Number of reptile species owned	**0.596**	<0.001	0.497	0.679
	Acquired the first reptile at age	−0.018	0.803	−0.158	0.123
	Reptile pet ownership duration (yrs.)	−0.009	0.898	−0.149	0.123
	Acquisition method	**0.401**	<0.001	0.276	0.513
	Satisfied with keeping reptile	**0.143**	0.040	0.002	0.278
Number of reptile species owned	Acquired the first reptile at age	−0.080	0.251	−0.218	0.061
	Reptile pet ownership duration (yrs.)	0.064	0.361	−0.077	0.203
	Acquisition method	**0.385**	<0.001	0.259	0.499
	Satisfied with keeping reptile	**0.184**	0.008	0.045	0.317
Acquired the first reptile at age	Reptile pet ownership duration (yrs.)	**−0.499**	<0.001	−0.598	−0.385
	Acquisition method	−0.072	0.301	−0.211	0.069
	Satisfied with keeping reptile	0.027	0.700	−0.114	0.167
Reptile pet ownership duration	Acquisition method	0.070	0.315	−0.071	0.209
	Satisfied with keeping reptile	**−0.181**	0.009	−0.313	−0.041
Acquisition method	Satisfied with keeping reptile	**0.177**	0.011	0.037	0.310

**Table 4:** Distribution of identified themes in open-ended justifications for reptile pet ownership, showcasing exemplars for each identified theme. Note: “O” in the “Participant” column denotes “Owner”.

	Total	Participant	Examples of Classified Responses (Translated)
Adoption	10	O53	Helping to take care of it when it was sick, gradually turned into a long-term relationship.
		O110	They are cute. And also, a lot of the turtles I look after over the years are rescued.
		O133	The turtle used to live in the company, but the company closed down and my colleague who was taking care of the animal was too busy, so I adopted the animal.
Aesthetic Appeal	77	O43	Cute and easy to take care of
		O79	Dinosaur-like
		O155	They look so cute
Ease of Care Expectations	38	O5	Children want to keep pets. As parents, we can try to satisfy that with our limited abilities and choose turtles that are easier to handle and raise.
		O10	Easy to manage compared with other animals
		O134	Easy to handle
Educational Value	5	O11	Parents buy animals for their child’s character development.
		O77	They’re unique, beautiful creatures that are misunderstood by the majority of the society. I hope that through keeping them, those around me can learn to appreciate these creatures.
		O113	My parents hoped I would develop a sense of responsibility raising pets when I was a child.
Emotional Connection	74	O8	I like to watch them hunting
		O141	Turtles have long life span. Wanted to have pets stay with me till I died.
		O192	I like them!
Family/Friend Influence	22	O36	Gift from a friend
		O60	Because my son wanted it
		O166	As a family activity with our son
Other	12	O20	Easy to take care of and cheap
		O94	They don’t take up much space, their habits are easy to master, and they don’t disturb the neighbors.
		O204	None

**Table 5:** Gender differences in reptile pet ownership based on the Kruskal–Wallis test results. Statistically significant correlations (*p* < 0.05) are indicated by bold font.

	Gender	Mean Rank	*p* Value
Number of pet reptiles	Female	97.14	**0.024**
	Male	115.86	
Number of reptile species owned	Female	98.45	**0.033**
	Male	113.31	
Acquired the first reptile at age	Female	111.19	**0.008**
	Male	88.56	
Reptile pet ownership duration (yrs.)	Female	93.96	**<0.001**
	Male	122.04	
Acquisition method	Female	100.27	0.191
	Male	109.77	
Satisfied on keeping reptile	Female	103.34	0.934
	Male	103.81	

**Table 6:** Respondent agreement with husbandry practices.

	Disagree	Somewhat Disagree	Somewhat Agree	Agree
	n (%)	n (%)	n (%)	n (%)
I think my reptile’s physical needs are met.	6 (2.9)	14 (6.8)	92 (44.7)	94 (45.6)
I think my reptile has enjoyed a habitat like its natural environment.	17 (8.3)	83 (40.3)	80 (38.8)	26 (12.6)
I think I have provided the enclosure in a size that is appropriate for my reptile.	6 (2.9)	37 (18.0)	97 (47.1)	66 (32.0)
I think I have adequately met the basking needs of my reptile.	3 (1.5)	15 (7.3)	79 (38.3)	109 (52.9)
I think I have adjusted the temperature properly for my reptile depending on the temperature change.	1 (0.5)	6 (2.9)	63 (30.6)	136 (66.0)
I think I have provided the diet that meets the nutritional needs of my reptile.	0 (0.0)	7 (3.4)	70 (34.0)	129 (62.6)
I think my reptile has received proper medical care.	1 (0.5)	12 (5.8)	70 (34.0)	123 (59.7)

**Table 7:** Distribution of adequate and inadequate husbandry practices in four key areas for reptile.

	Missing	Valid	Adequate	Inadequate
	n	n	n (%)	n (%)
Access to light	6	200	146 (73.0)	54 (27.0)
Temperature control	0	206	170 (82.5)	36 (17.5)
Hiding space	1	205	183 (89.3)	22 (10.7)
Regular check-up	7	199	103 (51.8)	96 (48.2)

**Table 8:** Distribution of normal and abnormal reptile behaviors including mean frequencies and standard deviations and the calculated reptile behavior score.

	Normal	Abnormal	Mean	Std. Deviation
	n (%)	n (%)		
Locomotor activity	189 (91.7)	17 (8.3)	1.08	0.276
Interaction with enclosure walls	145 (70.4)	61 (29.5)	1.30	0.458
Basking	160 (77.7)	46 (22.3)	1.22	0.417
Breathing	188 (91.3)	18 (8.7)	1.09	0.283
Actions towards humans	178 (86.4)	28 (13.6)	1.14	0.344
Actions in response to human presence or manipulation	175 (85.0)	31 (15.0)	1.15	0.358
Reptile behavior score	-	-	4.47	0.976

**Table 9:** Spearman’s rho correlation coefficients between reptile behavior and (i) demographic variables, (ii) reptile pet ownership ordinal variables. Statistically significant correlations (*p* < 0.05) are indicated in bold font.

Dependent Variable		Independent Variables	Correlation Coefficient (*r*)	*p* Value	95% Confidence Intervals	
					Lower	Upper
Reptile behavior score	(i)	Age	−0.014	0.845	−0.154	0.127
		Educational level	0.056	0.422	−0.085	0.195
		Working status	0.024	0.730	−0.117	0.164
		Household size	0.122	0.081	−0.019	0.258
		Living space area	**0.139**	0.046	−0.02	0.274
		Monthly household income	−0.001	0.994	−0.141	0.140
	(ii)	Number of pet reptile	0.081	0.245	−0.060	0.220
		Number of reptile species owned	0.110	0.114	−0.031	0.247
		Acquired the first reptile at age	−0.112	0.109	−0.249	0.029
		Reptile pet ownership duration	**0.200**	0.004	0.061	0.332
		Acquisition method	**0.162**	0.020	0.002	0.296
		Satisfied with keeping reptile	0.086	0.221	−0.056	0.224

## Data Availability

Data will be made available on request.

## Supplementary Materials

The following supporting information can be downloaded at: https://www.mdpi.com/article/10.3390/ani14121767/s1, Table S1: Questionnaire (Translated); Table S2: Justification of adequacy for four key husbandry practices in reptile care; Table S3: Justification of normal or abnormal reptile behavior.
