# Supplementary material for: Assessing the Current Landscape of Reptile Pet Ownership in Hong Kong: A Foundation for Improved Animal Welfare and Future Research Directions
Source: Animals (Basel). 2024 Jun 12;14(12):1767. doi: 10.3390/ani14121767 (PMC11201183; doi:10.3390/ani14121767)
Supplement: Supplementary file 1 [file animals-14-01767-s001.zip › Supplementary Materials (Table S1) - Questionnaire (Translated).docx]

**Table S1** – Full questionnaire

Dear reptile owners,

Thank you for taking part in the questionnaire! The aim for this survey is to have a better understanding on the relationship between owners’ social support and their behaviors on keeping reptile. This is an anonymous survey, all the information collected is used for academic research only and will not to disclosed. Thanks again for your kind assistance!

If you are interested in this research, please contract Swing Chan (postgraduate of Graduate Institute of Environmental Education, NTNU) by [61046020s@ntnu.edu.tw](mailto:61046020s@ntnu.edu.tw).

**Part 1 – General Information about the Owner and their pet history**

| Number of reptiles currently owned | 1  2 – 5  6 – 10  > 10 |
| --- | --- |
| Reptile(s) currently owned is/are: (please select all that apply) | Snakes  Lizards  Turtles |
| Gender | Male  Female  Others |
| Age | < 15  15 – 24  25 – 44  45 – 64  ≥ 65 |
| Educational level | Primary school or below  Junior secondary school  Senior secondary school  Tertiary (non-degree)  Tertiary (Degree)  Others: |
| Working status | Studying  Working  Unemployed  Retired |
| Monthly average household income | < HK$10,000  HK$10,000 – 19,999  HK$20,000 – 29,999  HK$30,000 – 39,999  HK$40,000 – 49,999  ≥ HK$50,000 |
| What is the total living area (sq. ft.) of your home? | < 100  100 – 300  301 – 500  501 – 800  > 800 |
| Number of people currently living together (including yourself)? | 1  2 – 4  5 – 7  ≥ 8 |
| Who do you live with? | Family  Friends  Roommates (e.g. student dormitories, shared rooms, etc.) |
| What is the attitude of your co-residents towards you keeping reptile? | Support  Not care  Against  Not know |
| At what age did you keep your first reptile? |  |
| How long (years) have you been keeping reptile(s)? | < 1  1 – 3  4 – 8  9 – 15  > 15 |
| What is the reason for you to keep reptiles? |  |
| How do you obtain your reptile(s)? (please select all that apply) | Purchase  Adoption  Gift from others  Wild found |
| Do you currently keep pet(s) other than reptile? | Yes  No |
| If yes, what other pet(s) do(es) you keep? (please select all that apply) | Dog  Cat  Birds  Arthropods e.g. insects, spiders, scorpions etc.  Others |

**Part 2 – Husbandry Questions**

Notes: If you have more than one reptile, please answer the following questions with the one you have kept for the longest time.

| The species of your reptile (ball python, gecko, iguana, leopard tortoise, red-eared slider etc.): |  |
| --- | --- |
| Recent photo of your reptile: (Optional) |  |
| I think I enjoy the experience of keeping reptile. | 1 – Disagree  2 – Somewhat disagree  3 – Somewhat agree  4 – Agree |
| I think my reptile has met its physical needs. | 1 – Disagree  2 – Somewhat disagree  3 – Somewhat agree  4 – Agree |
| I think my reptile has enjoyed a habitat like its natural environment. | 1 – Disagree  2 – Somewhat disagree  3 – Somewhat agree  4 – Agree |
| I think I have provided the enclosure in appropriated size that my reptile needed. | 1 – Disagree  2 – Somewhat disagree  3 – Somewhat agree  4 – Agree |
| I think I have adequately met the basking needs of my reptile. | 1 – Disagree  2 – Somewhat disagree  3 – Somewhat agree  4 – Agree |
| I think I have adjusted the temperature properly for my reptile depending on the temperature change. | 1 – Disagree  2 – Somewhat disagree  3 – Somewhat agree  4 – Agree |
| I think I have provided the diet that meets the nutritional needs of my reptile. | 1 – Disagree  2 – Somewhat disagree  3 – Somewhat agree  4 – Agree |
| I think my reptile has received proper medical care. | 1 – Disagree  2 – Somewhat disagree  3 – Somewhat agree  4 – Agree |
| Does your reptile have access to a source of sunlight or UVB light? (please select all that apply) | Yes, direct sunlight  Yes, sunlight through the window glass  Yes, UVA/ UVB lamp within the enclosure  Yes, UVA/ UVB lamp outside the enclosure  No  Don’t know |
| Have you monitor the enclosure temperature? | Continuous monitoring  Winter monitoring  No monitoring  Don’t know |
| Please specify the temperatures (℃) of the enclosures : |  |
| Have you provided hiding spaces for your reptile(s)? | Yes  No  Don’t know |
| Feeds you have provided for your reptile(s) include: (please select all that apply) | Commercial feed (pellets)  Fruit  Vegetables  Live food (e.g. crickets, grasshoppers, mice, worms, Dubai roach, fish etc.)  Others |
| Do you have regular check-up for your reptile(s) (e.g. fecal examination and deworming)? | Yes  No  Don’t know |
| Did your reptile(s) get sick before? | Yes  No  Don’t know |
| How did you deal with it? | Let the reptile heal itself  Buy medicine without consulting the vet  Vet consultation |

**Part 3 – Reptiles’ Behaviors**

Note: Does your reptile perform any of the following behaviors:

0 = No; 1 = Seldom; 2 = Often; 3 = Frequently

| Moves around in the enclosure investigating objects /people and exploring the environment. | \| 0 \| 1 \| 2 \| 3 \| \| --- \| --- \| --- \| --- \| |
| --- | --- | --- | --- | --- | --- |
| Attempts to push against, crawl up, dig under or round the enclosure barriers. | \| 0 \| 1 \| 2 \| 3 \| \| --- \| --- \| --- \| --- \| |
| Basks under the sunlight, UVB lamp or heat source with extended limbs and head. | \| 0 \| 1 \| 2 \| 3 \| \| --- \| --- \| --- \| --- \| |
| Mouth breathing with extended neck. | \| 0 \| 1 \| 2 \| 3 \| \| --- \| --- \| --- \| --- \| |
| Being aggressive towards people, e.g. biting or striking. | \| 0 \| 1 \| 2 \| 3 \| \| --- \| --- \| --- \| --- \| |
| Cloacal discharge (faeces or urine) or regurgitation in response to human presence or manipulation. | \| 0 \| 1 \| 2 \| 3 \| \| --- \| --- \| --- \| --- \| |

The questionnaire has been completed. You can click "Return" to view your answers. If there are no changes, please click "Submit".

Thank you for taking the time to fill it out!
