# Supplementary material for: Assessing the Current Landscape of Reptile Pet Ownership in Hong Kong: A Foundation for Improved Animal Welfare and Future Research Directions
Source: Animals (Basel). 2024 Jun 12;14(12):1767. doi: 10.3390/ani14121767 (PMC11201183; doi:10.3390/ani14121767)
Supplement: Supplementary file 1 [file animals-14-01767-s001.zip › Supplementary Materials (Table S2) - Justification of adequacy for four key husbandry practices in reptile care.docx]

**Table S2** – Justification of adequacy for four key husbandry practices in reptile care

|  | **Adequate/ Inadequate practice** | **Justification** |
| --- | --- | --- |
| Access to light source | Lizards & Turtles: Access to direct sunlight or UV lamp = adequate; no access to direct sunlight or UV lamp = inadequate  Snakes: Access to light = adequate; no access to light = inadequate | For lizards and turtles, UV or direct sunlight are essential for their survival and welfare. Failure to provide this resource results in metabolic bone disease (MBD), causing pain, muscle weakness, and fractures. Therefore, reptiles with direct access to either UV or sunlight were classified as having adequate husbandry practices, while those lacking both were classified as inadequate.  For snakes, the role of UV light in vitamin D sufficiency remains unclear. However, due to the presence of pituitary glands and photoperiodic regulation, this study classified reptiles without access to any light as having inadequate husbandry practices. |
| Temperature control | Regular or seasonal temperature control = adequate; no temperature control = inadequate | Reptiles are ectotherms, relying on external sources of heat to regulate their body temperature, with required ranges varying depending on the species and size. Recommended cool zone temperatures range from 21°C to 30°C, and hot zone temperatures from 30°C to 38°C (Boyer, 2009). Therefore, given the average temperature range in Hong Kong (14°C to 32°C, with occasional drops below 10°C in winter), a lack of temperature control was considered detrimental to thermoregulation and overall well-being in this study, potentially compromising the health and welfare of the reptiles. |
| Hiding space | Providing hiding space = adequate; not providing hiding space = inadequate | Within the reptile taxa investigated, the absence of appropriate hiding spaces was considered detrimental due to its impact on essential, likely phylogenetically ingrained behaviors. These behaviors, including camouflage and immobility, serve a vital function in predator evasion, thus promoting survival and potentially contributing to stress reduction and psychological well-being. Therefore, inadequate hiding opportunities compromise both the physical and potentially the psychological well-being of these reptiles. |
| Regular check-up | Providing regular check-up = adequate; not providing regular check-up = inadequate | While reptiles may instinctively conceal signs of illness to avoid predation, their ability to do so can delay necessary treatment when clinical signs eventually become evident. By implementing regular inspections, owners proactively safeguard the health of their reptiles and prevent potential crises. |
