# Supplementary material for: Assessing the Current Landscape of Reptile Pet Ownership in Hong Kong: A Foundation for Improved Animal Welfare and Future Research Directions
Source: Animals (Basel). 2024 Jun 12;14(12):1767. doi: 10.3390/ani14121767 (PMC11201183; doi:10.3390/ani14121767)
Supplement: Supplementary file 1 [file animals-14-01767-s001.zip › Supplementary Materials (Table S3) - Justification of normal or abnormal reptile behavior.docx]

**Table S3** – Justification of normal or abnormal reptile behavior

|  | **Survey Question** | **Normal/ Abnormal behavior** | **Justification** |
| --- | --- | --- | --- |
| Locomotor activity | Moves around in the enclosure investigating objects /people and exploring the environment. | No and seldom = abnormal; often and frequently = normal | Linked to the expression of exploratory and foraging behaviors. |
| Interaction with enclosure walls | Attempts to push against, crawl up, dig under or round the enclosure barriers. | No and seldom = normal; often and frequently = abnormal | Head pressing or rubbing against enclosure walls in reptiles can be associated with stress in captivity and interactions with transparent barriers. It may also be related to the inability to express innate behaviors, such as exploring, thermoregulating, and foraging. |
| Basking | Basks under the sunlight, UVB lamp or heat source with extended limbs and head. | No and seldom = abnormal; often and frequently = normal | Thermoregulatory behavior in ectotherms. |
| Breathing | Mouth breathing with extended neck. | No and seldom = normal; often and frequently = abnormal | Rapid open-mouthed breathing and neck extension with gasping movements are indicative of respiratory distress or hyperthermia. |
| Actions towards human | Being aggressive towards people, e.g. biting or striking. | No and seldom = normal; often and frequently = abnormal | Reptile aggression towards humans may be defensive in nature, and therefore potentially linked to fear-related motivations. |
| Actions in response to human presence or manipulation | Cloacal discharge (faeces or urine) or regurgitation in response to human presence or manipulation. | No and seldom = normal; often and frequently = abnormal | Cloacal discharge and regurgitation in reptiles can be elicited by fear or stress responses and serve as defensive mechanisms. |
